# A systematic review of artificial intelligence tools for chronic pulmonary embolism on CT pulmonary angiography

**DOI:** 10.3389/fradi.2024.1335349

**Published:** 2024-04-09

**Authors:** Lojain Abdulaal, Ahmed Maiter, Mahan Salehi, Michael Sharkey, Turki Alnasser, Pankaj Garg, Smitha Rajaram, Catherine Hill, Christopher Johns, Alex Matthew Knox Rothman, Krit Dwivedi, David G. Kiely, Samer Alabed, Andrew James Swift

**Affiliations:** ^1^ Department of Infection, Immunity and Cardiovascular Disease, The University of Sheffield, Sheffield, United Kingdom; ^2^ Faculty of Applied Medical Science, King Abdulaziz University, Jeddah, Saudi Arabia; ^3^ Respiratory Physiology Department, Sheffield Pulmonary Vascular Disease Unit, Sheffield, United Kingdom; ^4^ Department of Clinical Radiology, Sheffield Teaching Hospitals NHS Foundation Trust, Sheffield, United Kingdom; ^5^ Faculty of Medicine and Health Sciences, Norwich Medical School, University of East Anglia, Norwich, United Kingdom; ^6^ Faculty of Engineering, INSIGNEO Institute, Institute for in Silico Medicine, The University of Sheffield, Sheffield, United Kingdom; ^7^ Sheffield Biomedical Research Centre, National Institute for Health Research, Sheffield, United Kingdom

**Keywords:** artificial intelligence, deep learning, computed tomography pulmonary angiography (CTPA), chronic pulmonary embolism (CPE), chronic thromboembolic pulmonary hypertension (CTEPH)

## Abstract

**Background:**

Chronic pulmonary embolism (PE) may result in pulmonary hypertension (CTEPH). Automated CT pulmonary angiography (CTPA) interpretation using artificial intelligence (AI) tools has the potential for improving diagnostic accuracy, reducing delays to diagnosis and yielding novel information of clinical value in CTEPH. This systematic review aimed to identify and appraise existing studies presenting AI tools for CTPA in the context of chronic PE and CTEPH.

**Methods:**

MEDLINE and EMBASE databases were searched on 11 September 2023. Journal publications presenting AI tools for CTPA in patients with chronic PE or CTEPH were eligible for inclusion. Information about model design, training and testing was extracted. Study quality was assessed using compliance with the Checklist for Artificial Intelligence in Medical Imaging (CLAIM).

**Results:**

Five studies were eligible for inclusion, all of which presented deep learning AI models to evaluate PE. First study evaluated the lung parenchymal changes in chronic PE and two studies used an AI model to classify PE, with none directly assessing the pulmonary arteries. In addition, a separate study developed a CNN tool to distinguish chronic PE using 2D maximum intensity projection reconstructions. While another study assessed a novel automated approach to quantify hypoperfusion to help in the severity assessment of CTEPH. While descriptions of model design and training were reliable, descriptions of the datasets used in training and testing were more inconsistent.

**Conclusion:**

In contrast to AI tools for evaluation of acute PE, there has been limited investigation of AI-based approaches to characterising chronic PE and CTEPH on CTPA. Existing studies are limited by inconsistent reporting of the data used to train and test their models. This systematic review highlights an area of potential expansion for the field of AI in medical image interpretation.

There is limited knowledge of A systematic review of artificial intelligence tools for chronic pulmonary embolism in CT. This systematic review provides an assessment on research that examined deep learning algorithms in detecting CTEPH on CTPA images, the number of studies assessing the utility of deep learning on CTPA in CTEPH was unclear and should be highlighted.

## Introduction

Pulmonary hypertension is defined by elevated mean pulmonary artery pressure (mPAP) and results in right ventricular failure, with significant associated morbidity and mortality. Chronic thromboembolic pulmonary hypertension (CTEPH) is a subgroup of pulmonary hypertension in which the rise in mPAP is driven by repeated and/or large volume pulmonary embolism (PE) (1). Surgical pulmonary endarterectomy remains the gold standard treatment for CTEPH and is potentially curative. In patients for whom endarterectomy is unsuitable, alternative treatment options include endovascular pulmonary angioplasty and medical management with anticoagulation and pulmonary vasodilators. Early initiation of treatment is important for preventing disease progression and improving patient outcomes in CTEPH, but requires prompt diagnosis (2, 3).

Diagnostic delays are common for CTEPH, with an average of 14 months to diagnosis from the onset of symptoms (4). This can be attributable to the variability of clinical presentations and overlap of symptoms such as dyspnoea with a range of other potential causes (5). Right heart catheterisation remains the gold standard for diagnosis of pulmonary hypertension and CTEPH, but is invasive and not readily accessible in most centres. CT pulmonary angiography (CTPA) is well established as a non-invasive tool for the assessment of CTEPH. The modality is widely available, frequently performed for patients with cardiorespiratory symptoms and can provide information that assists with risk stratification, treatment decisions and prognostication (6). CTPA not only enables localisation and quantification of thromboembolic disease but can also yield biomarkers of disease severity (such as changes in pulmonary artery calibre and right ventricular morphology) and identify associated parenchymal lung changes (7, 8).

**Figure 1:**
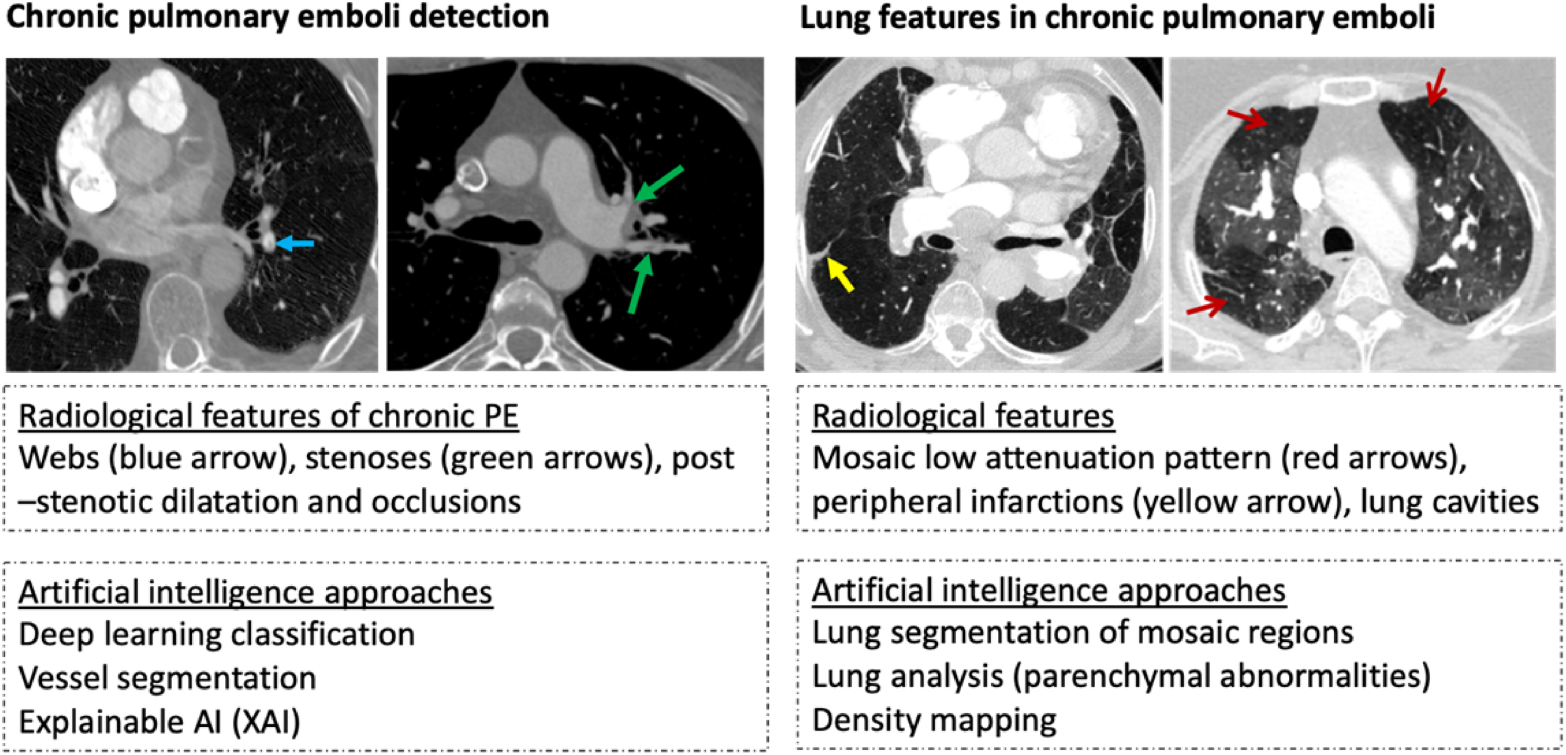
Example of radiological features and artificial intelligence approaches in chronic thromboembolic disease detection (images are from Sheffield institution on illustrating the diagnostic features of chronic pulmonary hypertension).

Automation of image identification tasks through AI offers potential improvements in diagnostic accuracy and efficiency. Various machine-learning methods have been applied to aid the detection and characterisation of acute pulmonary embolism on CTPA, with some tools licensed and in use as clinical decision aids (9–12). These include deep learning (DL) algorithms utilising neural networks—these comprise layers of interconnected artificial neurons, enabling algorithms to learn patterns and relationships from data and generate models that can be used to make decisions, such as image interpretation. Convolutional neural networks (CNNs) are frequently used in DL and are capable of performing imaging classification, segmentation, and detection of objects (13). Existing AI-based strategies for the evaluation of PE on CTPA have included image analysis to aid classification of disease and vessel segmentation. These techniques are still being developed and their incorporation into clinical practice will need more study, refining, and validation studies to assure their efficacy and accuracy (Figure 1). While there has been considerable interest in AI for the evaluation of acute PE, it is unclear to what extent AI has been used to evaluate CTEPH on CTPA. This systematic review aimed to identify and appraise the quality of studies presenting AI tools for the evaluation of chronic PE or CTEPH on CTPA.

## Methods

**Figure 2:**
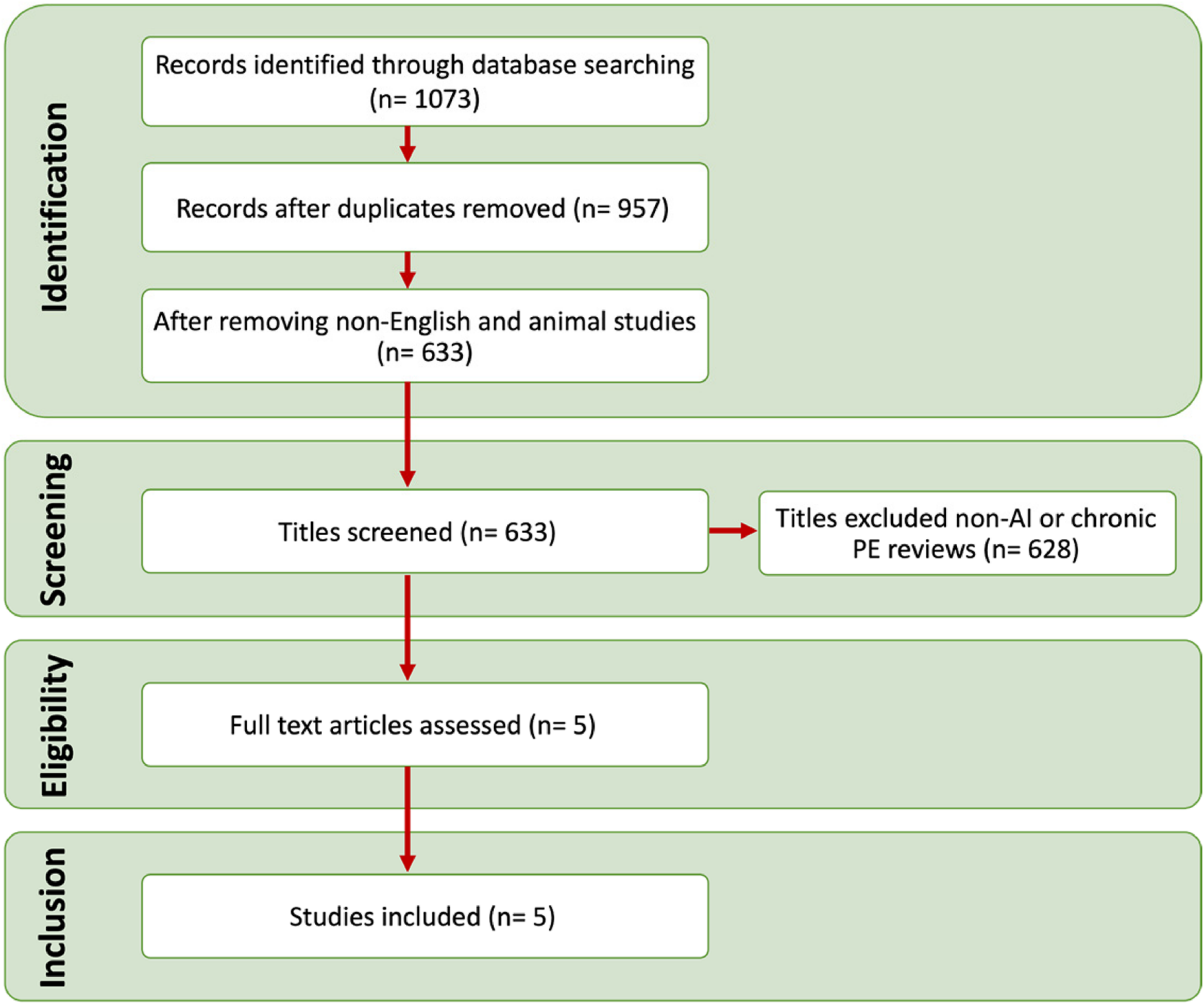
Process flow diagram for the inclusion and search steps.

The study was conducted in compliance with the Preferred Reporting Items for Systematic Reviews and Meta-Analyses (PRISMA) criteria (14). The study flow is presented in Figure 2.

### Eligibility criteria

Studies published in peer-reviewed journals from 2012 onwards were eligible for inclusion if they (1) presented or assessed any type of AI tool (2) for CTPA images (3) from participants with either confirmed chronic PE or CTEPH. Exclusion criteria included non-English-language publications, non-original research (such as reviews or letters) and animal or phantom studies.

### Search strategy

MEDLINE and EMBASE databases were searched on 11 September 2023. The search strategy is provided in the Supplementary Materials.

### Study selection and data extraction

Search results were screened for eligibility by two authors (LA and AS) independently by reviewing titles and abstracts using Rayyan Systematic Review Screening Software (15). Data were extracted from included studies using a standardised spreadsheet by two authors (LA and TA). Extracted data included study information (such as location, year and journal type), study design, data selection (such as number of participants, number of CTEPH cases and inclusion criteria), and the AI model being presented (such as validation and performance results). The quality of each included study was appraised by checking compliance with the individual criteria of the Checklist for Artificial Intelligence in Medical Imaging (CLAIM) (16), which were divided into four domains (17, 18).

## Results

Five studies were eligible for inclusion (Figure 2) and are summarised in Table 1 (19–23). Agreement of the studies with the criteria of CLAIM are presented in Table 2.

**Table 1:** An overview of the literature review papers that used CTPA to identify chronic pulmonary emboli using deep learning algorithms (NA = not applicable).

Study	No. Patients	Chronic PE	PE type	Setting for CTPA scan	Public (source)	AI type	Network	Outcomes
Vainio et al. (19)	50	25	Chronic	Multicentre	No	Segmentation	U-net-type, CNN	AUC 0.87
Khan et al. (20)	9,446	NA	Acute and chronic	Public	RSNA (Kaggle)	Classification	DenseNet201	AUC 0.90
Ma et al. (21)	7,279	NA	Acute and chronic	Public	RSNA (Kaggle)	Classification	3D CNN and 3D ResNet-18 model	Full cohort AUC 0.93/Chronic PE AUC 0.68
Vainio et al. (19)	Public 976 Local 78	Public 244 Local 26	Chronic	Local and public	RSPECT	Classification	CNN and DenseNet201	AUC 0.94 on local
Bird et al. (22)	161	51	CTEPH	Local	No	Segmentation	CNN	AUC 0.84

**Table 2:** Compliance with CLAIM checklist. Studies and the division of criteria into study description, dataset description, model description and model performance domains are shown below in (Blue/**✓** = Yes) to indicate compliance, (Red/x = No) for non-compliance and (Gray/ ─ = Not applicable).

	CLAIM checklist	Vainio et al. (19)	Khan et al. (20)	Ma et al. (21)	Vainio et al. (23)	Bird et al. (22)
Study description	Identification as a study of AI	**✓**	**✓**	**✓**	**✓**	**✓**
	Structured summary of study design, methods, results, and conclusions	**✓**	**✓**	**✓**	**✓**	**✓**
	Scientific and clinical background	**✓**	**✓**	**✓**	**✓**	**✓**
	Study objectives and hypotheses	**✓**	**✓**	**✓**	**✓**	**✓**
	Prospective or retrospective study	**✓**	**✓**	**✓**	**✓**	**✓**
	Study goal	**✓**	**✓**	**✓**	**✓**	**✓**
	Where the full study protocol can be accessed	x	**✓**	**✓**	**✓**	x
Dataset description	Data sources	**✓**	**✓**	**✓**	**✓**	x
	Eligibility criteria, symptoms, results from previous tests, inclusion in registry	**✓**	x	x	**✓**	**✓**
	Data pre-processing steps	**✓**	**✓**	**✓**	**✓**	x
	Selection of data subsets	**✓**	**✓**	**✓**	**✓**	**✓**
	Definitions of data elements	**✓**	x	x	**✓**	**✓**
	De-identification methods	**✓**	**✓**	**✓**	**✓**	x
	How missing data were handled	x	x	x	x	x
	Flow of participants	**✓**	**✓**	**✓**	**✓**	**✓**
	Sample size calculation	**✓**	**✓**	x	**✓**	x
	How data were assigned to partitions	**✓**	**✓**	**✓**	**✓**	**✓**
	Level at which partitions are disjoint	**✓**	**✓**	**✓**	**✓**	**✓**
	Demographic and clinical characteristics	**✓**	x	x	**✓**	**✓**
Ground truth reference standard	Definition of ground truth reference standard	**✓**	x	x	x	x
	Rationale for choosing the reference standard	x	x	x	x	x
	Source of ground truth annotations	**✓**	x	x	**✓**	x
	Annotation tools	**✓**	**✓**	**✓**	**✓**	x
	Inter- and intrarater variability	**✓**	x	x	**✓**	x
Model description	Detailed description of model	**✓**	**✓**	**✓**	**✓**	x
	Software libraries, frameworks, and packages	**✓**	**✓**	**✓**	**✓**	x
	Initialization of model parameters	**✓**	**✓**	**✓**	**✓**	x
	Details of training approach	**✓**	**✓**	**✓**	**✓**	x
	Method of selecting the final model	**✓**	**✓**	**✓**	**✓**	x
	Ensembling techniques, if applicable	x	**✓**	**✓**	**✓**	x
Model performance	Metrics of model performance	**✓**	**✓**	**✓**	**✓**	**✓**
	Statistical measures of significance and uncertainty	**✓**	**✓**	**✓**	x	**✓**
	Robustness or sensitivity analysis	**✓**	**✓**	**✓**	**✓**	**✓**
	Methods for explainability or interpretability	x	**✓**	**✓**	**✓**	**✓**
	Validation or testing on external data	x	x	x	**✓**	x
	Performance metrics for optimal model on all data partitions	**✓**	**✓**	**✓**	**✓**	x
	Estimates of diagnostic accuracy	**✓**	**✓**	**✓**	**✓**	**✓**
	Failure analysis of incorrectly classified cases	**✓**	**✓**	**✓**	**✓**	x
Other information	Study limitations	**✓**	x	**✓**	**✓**	**✓**
	Implications for practice	**✓**	**✓**	**✓**	x	x
	Registration number and name of registry	─	─	─	─	─
	Sources of funding	**✓**	**✓**	**✓**	**✓**	**✓**
	Overall % Compliance with CLAIM	85%	76%	76%	88%	50%

### Study 1—Vainio et al.

Vainio et al. (19) investigated the application of a 3D convolutional neural network (CNN) to identify hypoperfusion areas affected only by CTEPH from CTPA images. The overall compliance of the study with CLAIM was 85%. Compliance with the model description domain of CLAIM was 83%. The model comprised a U-net CNN (24) of twelve layers and three max-pooling/upsampling phases with skip connections and one output neuron with sigmoid activation in their 3D CNN layers. The Hounsfield Unit (HU) range was linearly shifted and scaled in order to resample and normalise the CTPA volumes. The patches totally outside the lung area were eliminated after the training data were partitioned into 32 × 32 × 32 voxel patches. Both learning rate adjustment and Dice loss optimisation were employed on manually labelled data to fine-tune the model and improve its accuracy during the validation process. The compliance with the dataset description criteria of CLAIM was 90%. The study used a dataset of 50 patients of which 25 (50%) had CTEPH. A positive ventilation–perfusion (V/Q) scan for chronic PE and a CTPA with evidence of chronic PE within 3 months without signs of acute PE were the inclusion criteria for the positive patients and confirmed with a right heart catheterisation. In all cases, radiological appearances of a parenchymal disease unrelated to CTEPH that involved more than two-thirds of the lungs were eliminated. The median age of all participants was 67 years and 62% were female. These were distributed into training, validation, and testing sets containing 48%, 12% and 40% of the data respectively. The study also showed complete compliance with the ground truth description criteria of CLAIM. Manual segmentation of affected regions on CTPA by one radiologist using ventilation-perfusion scan images was used as the ground truth. The compliance with the performance description criteria of CLAIM was 62%. The 3D CNN model performed segmentation of hypoperfused lungs with a reported area under the receiver curve (AUC) of 0.87. Failure analysis identified 63 independent false positive labels frequently attributed to beam hardening artefact.

### Study 2—Khan et al.

Khan et al. (20) presented a CNN model based on DenseNet201 (25) to classify a mixed cohort including acute and chronic PE on CTPA. The overall compliance of the study with CLAIM was 76%, including complete compliance with the model description criteria. The proposed model architecture for PE detection comprised an input module, a feature extractor module and a decision-making module. The feature extractor model, based on DenseNet201 (25), used densely connected convolutional blocks to extract rich hierarchical features from CT images, each comprising multiple convolutional layers, batch normalisation, and rectified linear activation functions (ReLU). The decision-making module took the extracted features and performed the final classification or decision-making process. This module consisted of intermediate dense and classification layers to produce the final prediction or decision regarding the presence or absence of PE. The compliance with the dataset description criteria of CLAIM was 66%. The study included 9,446 CTPA scans that were gathered from the RSNA-Kaggle public database (26) (available at: https://www.kaggle.com/c/rsna-str-pulmonary-embolism-detection). This dataset is classified into nine groups including undetermined PE, negative PE, right-side PE, Left-side PE, central PE, acute PE, chronic PE, and RV/LV ratio greater or less than 1. The dataset was annotated collaboratively by members of the RSNA and the Society of Thoracic Radiology and is a compilation of three previous datasets provided by the RSNA, with contributions from institutes in five countries (Canada, Brazil, Australia, Turkey and the USA). The 9,446 exams that make up the dataset made accessible on Kaggle have been divided in this study into two sets: a training set of 7,279 exams and a test set of 2,167 exams. While the dataset included acute and chronic PE cases, the proportion of patients in each group was not reported and there were no confirmed CTEPH cases. Compliance with the performance description criteria of CLAIM was 87%. The model achieved an overall accuracy of 88%, sensitivity of 88%, specificity of 89%, and AUC of 0.90 for all participants in the dataset. Although chronic PE subgroup was reported to have a 95% accuracy rate, with an AUC value of 0.95. The mean ROC curve—which averages the various ROC curves for each subgroup—had an AUC of 0.90.

### Study 3—Ma et al.

Ma et al. (21) also presented a model to identify PE on CTPA including acute and chronic PE in their dataset. The overall compliance of the study with CLAIM was 76% and the study also showed complete compliance with the model description criteria. The proposed approach entails a two-step pipeline: a 3D CNN model extracts a relevant feature sequence based on the surrounding area of slices, and a sequenced framework is used to produce study-level label predictions. The compliance with the dataset and ground truth description criteria were 58% and 20% respectively. The dataset used in this study was collected from the Kaggle competition RSNA STR Pulmonary Embolism Detection, which included 7,279 studies in total (26) (available at: https://www.kaggle.com/competitions/rsna-str-pulmonary-embolism-detection/data). The dataset includes labels at both the study and slice levels, with each slice including a label indicating if it contains any type of PE. However, the ground truth annotations used in their investigation were not defined. Compliance with the performance description criteria of CLAIM was 87%. The model performance in terms of PE identification had a reported sensitivity of 86% and specificity of 85%. For chronic PE cases, reported accuracy was 68%, sensitivity was 62% and specificity was 63%; however, the number of chronic PE cases was not reported in the paper, but it is available in the original public source.

### Study 4—Vainio et al.

Vainio et al. (23) developed a CNN tool with the aim of identifying and differentiating chronic PE from 2D maximum intensity projection (MIP) reconstructions of CTPA. The overall compliance with CLAIM was 88%, with complete compliance with the model description criteria. Deep learning-based lung segmentation was used to prepare the CTPA images for MIP reconstructions by removing high-intensity features. A base model trained on ImageNet was used as the foundation for their architecture (27). 11 MIP images were used as input data, with each image representing a different view of the same scan. Images were processed individually before being averaged and passed through a three-layer multilayer perceptron (MLP) using ReLU activations. During training, alpha dropout and batch normalisation techniques were applied to the MLP layers. Transfer learning was also used, allowing the model to leverage pre-trained neural network architectures. Compliance for both the dataset and ground truth description domains of CLAIM was 82%. The publicly available RSNA-STR Pulmonary Embolism CT (RSPECT) dataset was used for training. This was divided into two experiments (28). The first included 755 CTPA studies, focusing on discriminating between patients with chronic PE (RV/LV ratio ≥1) and a control group composed of patients with acute PE (RV/LV <1) and those with negative PE examinations. Experiment 2 used the same groups as Experiment 1 but did not apply the RV/LV criterion, resulting in 976 CTPA scans. Additionally, a local dataset was utilised for validation and testing, consisting of 78 cases in total (26 for each of chronic PE, acute PE, and no PE).

The MIP images were modified by a radiologist manually selecting optimal colour and opacity transfer functions. Following the appearance adjustments, the researchers conducted visual inspections of the images to ensure that they accurately represented the required features and characteristics. Compliance with the performance description domain of CLAIM was 75%. In Experiment 1, DenseNet-121 with random 3-degree 2D rotations yielded the best performance, achieving an AUC of 0.70. Experiment 2, which used larger CTPA volumes for training and omitted RV/LV-based exclusion, resulted in slightly lower performance. An ensemble model was introduced, leading to a modest increase in balanced accuracy. The local dataset outperformed the public dataset significantly, with an AUC of 0.87 compared to 0.79. In the third stage, using a local dataset of 78 cases for model selection and testing led to an AUC of 0.94 and an overall accuracy of 0.89.

### Study 5—Bird et al.

This study aimed to evaluate a new automated method for quantifying hypoperfusion on dual energy CTPA to help in assessing the severity of CTEPH (22). An established DL CNN model for lung segmentation was utilised to automatically segment hypoperfused lung volume, effectively removing extraneous thoracic anatomy and delineating lobar boundaries. This involved processing CT images alongside iodine-water images to compute and measure the proportion of hypoperfused pixels within each lobe (29). The overall compliance with CLAIM was 50%. The study referenced but did not provide any description of the CNN model (29). Similarly, there was limited description of the datasets used, with 66% compliance with the dataset description criteria. The data study used a locally obtained data from, 51 CTEPH patients and 110 normal CTPA scans were retrospectively analysed. The model automatically isolated parenchymal iodine values to delineate hypoperfusion areas and calculate hypoperfused lung volume. Compliance with the performance description criteria was 62%. The model showed that global hypoperfused lung volume distinguished CTEPH patients from controls with 0.84 AUC and 90% sensitivity cutoffs, and correlated positively with hemodynamic severity and changes after surgical treatment. The study concluded that automated quantification of hypoperfused areas in CTEPH patients from dual energy CTPA may assist in clinical evaluation, especially in cases involving segmental-level disease.

## Discussion

The application of AI to CTPA interpretation in the context of chronic PE and CTEPH is appealing. AI tools have the potential to aid the detection of cardiovascular and lung parenchymal changes that are important for diagnosis, risk stratification, prognostication and treatment decisions in chronic PE. Previous studies suggested that a lack of sensitivity for PE detection may affect radiologists' interpretation, which ranged from 66% to 87% (30, 31). AI tools for the identification of chronic PE could assist the accuracy and efficiency of CTPA interpretation by radiologists, such as by highlighting areas of potential concern for closer scrutiny. This is particularly relevant given that the changes in chronic PE may be incidental or subtle on imaging.

This systematic review aimed to identify and appraise existing studies presenting AI tools for CTPA in chronic PE patients. Five studies were eligible for inclusion, identifying a significant gap in the field. Study quality was evaluated using compliance with the criteria of CLAIM, an established structured checklist designed to aid the presentation and interpretation of studies presenting AI approaches. All five studies share a common focus on the application of deep learning techniques, particularly CNN algorithm for the detection and diagnosis of PE, using texture segmentation of the lung parenchyma without direct assessment of the pulmonary arteries. One study divided their analysis into several phases and made use of both public RSPECT and local datasets. Vainio et al. (19) demonstrated that segmentation of the hypoperfused lungs was carried out using the CNN model resulting in an AUC of 0.87 for detecting chronic PE. Khan et al. (20) reported that the model achieved an overall AUC of 0.90, for all participants in the dataset. Ma et al. (21) showed promising PE detection ability whether acute or chronic, with a window-level AUC of 0.93. Vainio et al. (23), showed that a relatively limited local dataset for model selection and testing resulted in an AUC of 0.94, indicating efficacy in diagnosing chronic PE. However, there is a potential risk of overfitting, increased variability, and uncertainty associated with using a small dataset, as it may not fully capture the variability and complexity of the underlying population.

### Models

The included studies provided reliable descriptions of their respective deep learning CNN models. Khan et al. (20) explored the use of DL CNN algorithms in computer-aided diagnosis of PE. The models were trained on a large dataset of CT scans and employed advanced techniques for feature extraction and classification to improve the accuracy of PE diagnosis. DenseNet201 is a type of neural network that employs densely connected convolutional blocks; each layer receives direct inputs from all preceding layers, resulting in enhanced feature propagation and reuse throughout the network (25). This connectivity pattern allows for better information flow and gradient propagation, potentially improving the model's ability to learn complex patterns and features relevant to PE detection. The dense connections can lead to increased memory requirements, as the outputs of all preceding layers need to be stored for gradient computation during backpropagation. DenseNet201 is a highly expressive model with a large number of parameters. In some cases, this can increase the risk of overfitting, especially if the dataset is small or not diverse enough.

In the study by Vainio et al. (23), the model to detect chronic PE used transfer learning from a previously trained ImageNet model (27), analysed eleven MIP images, combined the image characteristics and processed them using an MLP with ReLU activations. The use of 2D MIP reconstructions for training an AI tool on CTPA images as opposed to using other approaches has benefits in terms of standardisation, computational efficiency and ease of use. However, it comes with the risk of missing essential 3D information and location-based context, which can have an influence on the tool's diagnostic accuracy. In their earlier study, Vainio et al. (19) focused on the evaluation of a 3D CNN for the detection of hypoperfusion in patients with CTEPH. They investigated the feasibility and effectiveness of using a 3D CNN architecture to analyse CTPA images and identify regions of hypoperfusion in the lung. Ma et al. (21) presented a multitask DL approach for the detection and identification of PE, with a CNN architecture capable of simultaneously performing multiple tasks related to PE diagnosis, such as segmenting affected lung regions, classifying the severity, and providing a score for diagnosis. The multi-task deep learning model was trained on a diverse dataset and is expected to improve the efficiency and accuracy of PE detection and identification, as the multitask learning strategy enabled the model to train and execute both PE detection and identification tasks simultaneously. This could lead to more efficient and streamlined predictions by leveraging shared information between the tasks. The model may be able to identify frequent patterns and features for both identifying and detecting PE, improving the predictions made by the model's overall resilience and accuracy. However, multitask learning can be challenging if the tasks have conflicting or unrelated objectives. If the tasks have different characteristics or require distinct feature representations, jointly training them may hinder the performance on individual tasks. Every task needs a substantial amount of labelled data. If one task has a considerably smaller dataset or lacks labelled data, the performance of both tasks might be affected. This can result in a more complex model architecture, which may increase the risk of overfitting and require more computational resources for training and inference. While Bird et al. (22) study focused less on developing and more on validating or assessing the algorithm. This approach can save space and reduce redundancy in the paper, authors must ensure that they follow ethical and academic standards by accurately crediting the original developers of the CNN model and providing readers with enough information to understand its implementation and performance in their study.

Overall, these papers share a common objective of leveraging DL techniques to improve the detection and diagnosis of PE from CTPA images. Some studies failed to assess the performance of their model on external datasets, potentially limiting their validity. However, only one study, conducted by Vainio et al. in 2023, addressed this limitation by testing their model on external datasets. Vainio et al. (23) is the only study that reports a validation step during the development of their model. Assessing performance on validation data enables model fine-tuning (such as through hyperparameter optimisation) and the identification of potential issues (such as overfitting) prior to final model selection and testing. A lack of validation may limit the overall performance of these models. This raises concerns about the model's ability to function effectively and consistently in clinical practice settings. Positively, all of the research examined the causes of model underperformance and offered failure analyses of cases that were misclassified—an important step in ensuring validity.

### Datasets

Descriptions of the datasets were less consistent, potentially limiting the interpretation of model performance. Each study did report their data sources, apart from Bird et al. (22). Public datasets from the RSNA were used in three of the studies (20, 21, 23). The use of public datasets offers ease of access to data, improves study transparency, and helps comparison between models. Using diverse datasets results in a bigger annotated dataset with a broad range of samples from around the world. However, model performance may be restricted by the availability of data elements or variables in public datasets, which were not mentioned in two publications (20, 21). These two studies used publicly available Kaggle datasets for the detection of acute and chronic PE. Although the proportions of acute and chronic PE cases are available from the original data source, these were not stated by the studies themselves, limiting their transparency—it is best practice for publications to provide all relevant clinical characteristics regardless of whether they can be accessed elsewhere (17, 18). Public datasets utilised in the studies may not have had all the elements or features required for an accurate PE diagnosis, which may limit the model's ability to identify the spectrum of abnormalities related to PE. Variations in data quality may also affect model performance by affecting generalisability across datasets with distinct characteristics. While age and sex are important demographic factors, they should be complemented with additional information—such as the severity of disease or presence of comorbidities—to provide a more comprehensive understanding of generalisability in clinical populations. We observed that two out of five studies (20, 21) lacked information on the demographics of these patients as well as the percentages of patients with various diseases. Studies should not assume that their audience is already acquainted with public datasets, and study methodology must be explained in sufficient detail to allow correct reproduction of the results.

Image annotations in public datasets may also be limited and restrict how the data can be used. For example, Ma et al. (21) demonstrated that certain labels are directly taken by others and cannot be modified or changed. As a result, they do not account for the possibility of an inconsistent relationship between the labels and the predicted study-level labels in their model. To increase the generalisability of AI models being trained, different data sets, such as retrospective and prospective data sets, might be combined. We found that one of the studies in this review was created by combining three other datasets that RSNA had previously provided with contributions from five more countries and institutions (20). The three public dataset studies did not include the description of the ground truth reference standard and the rationale for choosing the reference standard in their paper, although this information is accessible from the public source. Vainio et al. (23) trained their model using the publicly available RSPECT dataset, but validated and tested the model using an external local dataset. Testing model performance on external data is important for ensuring generalisability and is an important consideration for clinical translation of AI tools. However, it is worth noting that only 78 cases—including only 26 cases of chronic PE—were included in this external dataset, which limits interpretation of the model's performance. It is important to carefully analyse the context and objectives of the AI model when deciding to use public data for training and local data for testing and model selection. While combining different datasets can have benefits like increased variety and generalisation, it also has drawbacks including inconsistent data, bias, and small local sample sizes. Improvements are needed in public datasets to address the lack of flexibility and adaptability in the labelling process, providing detailed information about datasets characteristics, and data augmentation applied. Some of these limitations may be addressed, and the robustness and dependability of the model can be improved, by making an effort to collect more comprehensive and diverse local data or by working with several healthcare facilities.

### Performance

The Vainio et al. (19) and Bird et al. (22) studies were the only papers that evaluated hypoperfusion areas affected by CTEPH specifically on CTPA images using a DL model. Vainio et al. (19) did not specifically look into the pulmonary vessels, rather evaluated the secondary effects on lung parenchyma. The AUC curve was solely used in this study to assess the model's performance. The performance of the 3D CNN model could be evaluated using various metrics such as sensitivity, specificity, accuracy, and area under the receiver operating characteristic curve (AUC-ROC). These metrics provide insights into the model's ability to correctly identify positive and negative cases of hypoperfusion. There are several metrics available for evaluating DL models. Using merely a subset may offer a misleading overview of a model's real performance, resulting in unexpected findings when applied in a clinical setting. Therefore, it's crucial to combine several measures and analyse performance comprehensively (32). However, the study might have compared the performance of the 3D CNN model with existing methods used for detecting hypoperfusion in CT pulmonary angiography. This could involve comparing the AUC to determine if the 3D CNN outperforms or is comparable to other approaches. The study demonstrated the feasibility of using a 3D CNN for the automated detection of hypoperfusion on CTPA images in patients with CTEPH. Furthermore, the results indicated that CNNs were able to automatically support radiologists in diagnosing and treating patients with chronic PE. Vainio et al. (23) assessed the CNN model in several phases. The model performed inconsistently across the public and local datasets, with the local dataset producing noticeably better outcomes. The model's ability to identify chronic PE was greatly enhanced by the application of a locally optimised ensemble model, challenging model selection techniques, and the local test dataset. The model improved in cross-validation model selection, sensitivity to data augmentation, and performance on the local dataset. However, there were differences in performance, as well as the effect of different training methods. The use of a limited local dataset for early stopping presents issues regarding overfitting. However, the other two studies by Khan et al. (20) and Ma et al. (21) evaluated PE in general (acute and chronic) and RV/LV ratio using CNN on CTPA for classification. Nonetheless, they omitted to present the number of acute and chronic cases, which limits interpretation. Khan et al. (20) trained an AI model without applying a validation set, which can have a number of consequences for the model's performance and reliability. Assessing performance using a validation set prior to formal testing is important for confirming that a model operates properly on unseen data and enables further refinement prior to finalisation. The lack of a validation step may limit understanding of model generalisability, increase the risk of overfitting, restrict hyperparameter optimisation and impede model selection. Finally, this study analyses the possible advantages of IoMT-enabled computer-aided diagnostics for PE classification. Gradient-weighted class activation mapping (Grad-CAM) was used by Ma et al. (21) to improve the interpretability of the AI model, although this only applied to the first phase of training rather than going over the sequential model's parameters in the second training phase. The exclusion of Grad-CAM during the second phase of training may limit the interpretability of the model's updated parameters. The overall results demonstrate that their model was accurate in detecting and classifying PE and has the potential to enhance acute PE diagnosis. While in chronic PE, the model does not perform effectively although they omitted to give the number of chronic PE cases.

Our systematic review has limitations. The eligibility criteria focused on chronic PE detection using AI on CTPA images; studies may have been missed if they had not clearly identified the presence of chronic PE in their datasets. Unpublished research and non-English language studies were not included. Despite the inclusion of conference abstracts within the eligibility criteria, the number of included studies was low, preventing formal meta-analysis of model performance.

## Conclusion

This systematic review identified five existing studies presenting AI tools for CTPA interpretation in patients with chronic PE or CTEPH. All studies presented DL CNN approaches to the assessment of lung parenchyma, with variable performance. Assessment of studies using CLAIM identified overall reasonable reporting of AI model design and training, but inconsistent reporting of the datasets used, limiting their transparency. This study highlights an area of potential expansion for the field of AI in medical imaging.

## Data Availability

The original contributions presented in the study are included in the article/Supplementary Material, further inquiries can be directed to the corresponding author.
