# Supplementary material for: A systematic review of artificial intelligence tools for chronic pulmonary embolism on CT pulmonary angiography
Source: Front Radiol. 2024 Apr 9;4:1335349. doi: 10.3389/fradi.2024.1335349 (PMC11035730; doi:10.3389/fradi.2024.1335349)
Supplement: Supplementary file 1 [file Datasheet1.docx]

**Supplementary material**

**Database search strategy**

Ovid (MEDLINE and EMBASE) Search Strategy:

1. (Convolutional OR neural) ADJ1 network
2. (Deep or supervised or unsupervised or machine) AND learning
3. AI OR (Artificial ADJ1 intelligence) OR Algorithm
4. Machine Learning MESH
5. 1 OR 2 OR 3 OR 4
6. (Pulmonary emboli) OR PE OR CTEPH or CTED
7. Lung Embolism MESH
8. 6 OR 7
9. (CT pulmonary angiography) or CTPA
10. Computer Tomography MESH
11. 9 OR 10
12. 5 AND 8 AND 11
